# Clinical and molecular correlates of limbic age-related TDP-43 encephalopathy (LATE) ^18^F-FDG-PET pattern in amnestic mild cognitive impairment

**DOI:** 10.1007/s00259-025-07395-9

**Published:** 2025-06-13

**Authors:** Cecilia Boccalini, Luisa Knappe, Gregory Mathoux, Debora Elisa Peretti, Federica Ribaldi, Francesca B. Pizzini, Linjing Mu, Max Scheffler, Giovanni B. Frisoni, Valentina Garibotto

**Affiliations:** 1 Laboratory of Neuroimaging and Innovative Molecular Tracers (NIMTlab), Geneva University Neurocenter and Faculty of Medicine, University of Geneva, Geneva, Switzerland; 2 Division of Nuclear Medicine and Molecular Imaging, Geneva University Hospitals, Geneva, Switzerland; 3 Geneva Memory Center, Department of Rehabilitation and Geriatrics, Geneva University Hospitals, Geneva, Switzerland; 4 LANVIE (Laboratory of Neuroimaging of Aging (LANVIE), University of Geneva, Geneva, Switzerland; 5 Department of Engineering for Innovation Medicine, University of Verona, Verona, Italy; 6 Institute of Pharmaceutical Sciences, ETH Zurich, Zurich, Switzerland; 7 Division of Radiology, Geneva University Hospitals, Geneva, Switzerland; 8 CIBM Center for Biomedical Imaging, Geneva University Hospitals, Geneva, Switzerland

**Keywords:** Fluorodeoxyglucose positron emission tomography, Limbic age-related TDP-43 encephalopathy, Mild cognitive impairment, Alzheimer’s disease

## Abstract

**Purpose:**

This study aimed to test the ability to visually detect the characteristic medial temporal and limbic hypometabolic pattern of limbic age-related TDP-43 encephalopathy (LATE) in ^18^F-FDG-PET of patients with amnestic mild cognitive impairment (aMCI) and evaluate its prognostic value.

**Methods:**

We included 70 patients with aMCI who underwent ^18^F-FDG-PET, amyloid-PET, tau-PET, and structural MRI, as well as baseline and follow-up cognitive evaluation. ^18^F-FDG-PET scans were analyzed visually with single-subject maps, categorized as normal, Alzheimer's disease (AD)-like, LATE-like, or other neurodegenerative diseases, while blinded from other data. Clinical and biomarker features as well as cognitive trajectories were compared between groups.

**Results:**

25 scans were classified as normal, 25 as AD-like, 12 as LATE-like, and 8 as others. Patients with AD-like patterns were younger, had lower MMSE scores, inferior-to-medial temporal metabolism ratio, and greater hippocampal atrophy and cortical tau load than subjects with normal scans. Patients with LATE-like patterns had lower MMSE scores and more hippocampal atrophy than subjects with normal scans. Patients with LATE-like patterns were significantly older, had greater inferior-to-medial temporal metabolism ratio, greater amygdalar atrophy, and lower cortical tau load than subjects with AD-like patterns. Only subjects classified as AD-like showed a faster cognitive decline than negative scans.

**Conclusion:**

LATE-like hypometabolic pattern in aMCI can identify a subgroup of subjects distinct from AD and controls in terms of clinical severity, medial temporal atrophy, cortical tau load, and cognitive decline, supporting the utility of ^18^F-FDG-PET as a biomarker that provides inferential support for the specific detection of LATE.

**Supplementary Information:**

The online version contains supplementary material available at 10.1007/s00259-025-07395-9.

## Introduction

Mild cognitive impairment presenting with amnestic syndrome (aMCI) and amyloid positivity is usually considered due to Alzheimer’s disease (AD) [1], although a relevant proportion of subjects shows an overall slow clinical progression and 15 to 30% of clinically diagnosed AD do not meet neuropathologic criteria for AD [2]. Pathological studies linked these clinical cases, with late-onset aMCI with a slow disease course, to limbic-predominant age-related TDP-43 encephalopathy (LATE), either with or without hippocampal sclerosis [3]. LATE is a prevalent neurodegenerative pathology in aging populations that clinically mimics AD in persons of advanced age, with a significant and generally under-recognized impact on public health [3, 4]. LATE frequently co-occurs with AD [5] or may present as an independent pathology [6]. In either case, it poses a significant diagnostic challenge due to overlapping clinical features. Typically it shows a more memory-predominant profile with sparing of executive functions and a slower clinical progression compared to AD [3]. However, differentiation between LATE and AD is only possible postmortem as no validated in vivo biomarkers for TDP-43 pathology exist.

Among biomarkers that may provide inferential support for the presence of LATE neuropathologic change (LATE-NC) [7], ^18^F-fluorodeoxyglucose (^18^F-FDG) positron emission tomography (PET) has consistently informed patterns of hypometabolism suggestive of LATE. Hypometabolism in medial temporal structures has been described in pathologically confirmed patients with MCI and dementia due to TDP-43 pathology and/or hippocampal sclerosis [8, 9]. However, no studies have yet tested the ability of the visual assessment to detect patterns of hypometabolism suggestive of LATE at the individual level and their clinical relevance.

Given the promising role of ^18^F-FDG-PET in providing probabilistic support for a clinical diagnosis of LATE, this study aims to verify the ability to visually detect the LATE hypometabolic pattern in prodromal aMCI at the single subject level in a real clinical setting and to investigate its clinical usefulness. Since targeted treatments in amyloid positive aMCI became available, an early and correct diagnosis is even more urgent.

## Materials and methods

### Participants

70 subjects with aMCI were retrospectively included from the Memory Center of Geneva University Hospitals (HUG). Each subject underwent the Memory Center’s routine clinical workup, including clinical and neurological assessment, neuropsychological testing, and MRI. Additional procedures, such as amyloid-PET, tau-PET, and ^18^F-FDG-PET have been performed if deemed clinically useful, or in the context of research projects. Inclusion criteria were: (i) aMCI diagnosis according to Petersen's criteria [10]; (ii) Mini-Mental State Examination (MMSE), (iii) an ^18^F-FDG-PET scan, (iv) a tau-PET scan, (v) an amyloid-PET scan, and (vi) a structural MRI, all within a year from ^18^F-FDG-PET. 53 out of 70 subjects had a follow-up MMSE at 2.3 ± 1.2 years from the initial one. Neuropsychological testing included the Free and Cued Selective Reminding Test (FCSRT) 16-item version with immediate recall, delayed free and delayed total recall to assess episodic memory; Trail-Making Test (TMT) part A and part B to assess executive function; Semantic and Phonemic Fluency to assess executive abilities-working memory-language; and Digit Span (from WAIS-IV) to assess working memory.

The local ethics committee approved the imaging studies, which were conducted under the principles of the Declaration of Helsinki and the International Conference on Harmonization Good Clinical Practice. Each subject or their relatives provided voluntary written informed consent to participate in the studies.

### Imaging acquisition

MRI was performed at Division of Radiology at HUG using a 3 Tesla scanner (Magnetom Skyra, Siemens Healthineers, Erlangen, Germany), equipped with a 20- or 64-channel head coil. The following acquisition parameters were used: repetition time [TR] = 1810–1930 ms, echo time [TE] = 2.19–2.36 ms, field of view = 256 × 256 mm, flip angle = 8◦, slice thickness = 0.9–1 mm, matrix size = 288 × 288 pixels, or 256 × 230 pixels.

All PET scans were performed at the Nuclear Medicine and Molecular Imaging division at HUG with Biograph128 mCT, Biograph128 Vision 600 Edge, Biograph40 mCT, or Biograph64 TruePoint PET scanners (Siemens Medical Solutions, Malvern, PA, USA). All scanners were thus from the same vendor and generation, harmonized regarding their performance and reconstructions, and cross-calibrated. Data was acquired in list mode for all tracers and reconstructed using 3D OSEM, corrected for randoms, dead time, normalization, scatter, attenuation, and sensitivity, and averaged after motion correction. A 2-mm Gaussian filter at full width at half maximum (FWHM) was applied resulting in images with a 400 × 400 pixels matrix with 1.01 mm^3^ isotropic voxels.

^18^F-FDG-PET acquisition was performed according to the European Association of Nuclear Medicine (EANM) guidelines [11]. Subjects were injected with 203 ± 15 MBq of [^18^F]FDG via a venous cannula, eyes open in a dimly lit room [^18^F]flortaucipir (^18^F-AV1451) was used for the tau-PET scans. The tracer was synthesized at the Center for Radiopharmaceutical Sciences in ETH Zurich, Switzerland, under license from the intellectual property (IP) owner (Avid subsidiary of Lilly, Philadelphia, PA, USA). Subjects received 180 ± 50 MBq of ^18^F-AV1451, with image acquisition performed 75 min after injection (acquisition time 30 min). Each emission frame was reconstructed in 6 × 5-min frames. Amyloid-PET images were acquired using [^18^F] florbetapir (FBP) or [^18^F] flutametamol (FMM). FBP late images were acquired 50 min after the intravenous administration of 210 ± 18 MBq (3 × 5-min image frames). FMM late images were acquired 90 min after the intravenous administration of 166 ± 16 MBq (4 × 5-min image frames).

### Imaging processing

T1-weighted MRI images were segmented using Freesurfer (v.7.0; surfer.nmr.mgh.harvard.edu/) and hippocampal (HPV) and amygdala volumes were extracted. Medial temporal atrophy (MTA) was visually assessed according to the established score [12] from 0 (no atrophy) to 4 (significant atrophy). Amygdalar atrophy was visually assessed on a 3-level scale (no atrophy; mild/moderate atrophy; severe atrophy) following a standardized approach [13].

PET image processing was performed using Statistical Parametric Mapping (SPM12, Wellcome Trust Centre for Neuroimaging, London, UK), running in MATLAB R2018b Version 9.5 (MathWorks Inc., Sherborn, MA, USA). 3D T1 MRI images were aligned to a reference plane passing through the anterior commissure, segmented into grey matter, white matter, and cerebrospinal fluid tissue compartments, and normalized to the Montreal Neurologic Institute (MNI) space using tissue probability maps. All PET images were aligned to the subject’s respective T1 MRI scan and normalized to the MNI space using the transformation matrix that was generated during the registration of the MRI images to the standard space.

^18^F-FDG standardized uptake values (SUV) were extracted within regions from AAL 3 atlas [14]. SUV ratio (SUVR) was calculated by normalizing the uptake to the mean value of the pons and cerebellar vermis together as the reference region. Intensity-normalized PET images were saved for further single-subject voxel-wise analyses. The ^18^F-FDG-PET inferior-to-medial temporal (IMT) ratio was derived by dividing SUVR values from the inferior temporal lobe by the average of SUVR values from the amygdala and hippocampus [15].

Tau SUVR was calculated from the regions of interest (ROIs) of the AAL atlas 3 using the cerebellar crus as a reference region. We calculated the global SUVR from a global region including the parahippocampal gyrus, lateral occipital cortex, inferior temporal cortex, and amygdala [16], and in Braak stages [17]. Amyloid SUVR was calculated using the whole cerebellum as the reference region. SUVR was extracted from the Centiloid volume of interest (VOI) and converted into Centiloid units as recommended by Klunk [18]. A Centiloid value of 19 was used as the cutoff point to define amyloid positive versus negative individuals [19].

### ^18^F-FDG-PET visual rating

^18^F-FDG PET uptake distribution images together with the correspondent SPM single-subject maps (as semiquantitative support) were visually rated separately and randomly by two nuclear medicine physicians. Each single-subject hypometabolic map was obtained by applying a voxel-wise two-sample t-test with an ^18^F-FDG-PET internal database of healthy controls (HC; *N* = 43; age = 74 ± 8.85) in SPM12, including age as a covariate [20]. The HC dataset was defined as amyloid-negative based on PET and cognitively unimpaired (MMSE ≥ 28). The p-value of the single-subject hypometabolism maps was set at *p* < 0.05 uncorrected at the cluster level, with a cluster-forming threshold of *n* = 100 voxels. Two nuclear medicine physicians (GM and LK) blinded to clinical information classified the single-subject SPM hypometabolic maps into hypometabolism patterns suggestive of neurodegenerative conditions [9, 21, 22] or excluding the presence of significant neurodegeneration. In case of doubtful classification, the scans were reassessed using the commercial software Database Comparison for Syngo.via, provided by Siemens, Healthineers, Erlangen, Germany. This method includes its own database of normal subjects according to different age groups. In cases where there was disagreement among the physicians’ readings, a consensus reading was obtained involving also a third expert rater (VG) and then used for statistical analyses aiming at validating the visual classification against other clinical and biomarker data. Figure S1 reported a flowchart of the study.

### Voxel-wise analysis

Voxel-wise SPM t-test analyses, controlling for age and MMSE, were applied to compare visually defined groups to assess brain metabolism at the group level and voxel-wise differences in metabolism. Specifically, AD-like and LATE-like groups were compared to negative scans to investigate the group hypometabolic pattern and between each other to see voxel-wise metabolic differences between LATE-like and AD-like groups. The statistical threshold was set at *p* = 0.005, FWE-corrected for multiple comparisons. Only clusters containing more than 100 voxels were deemed to be significant.

### Statistical analysis

Demographic and clinical characteristics were compared between the groups based on ^18^F-FDG-PET patterns classification using the Kruskal-Wallis rank sum test for continuous variables and a proportion test for categorical variables. ANCOVA tests were applied to compare biomarkers data using age and MMSE as nuisance variables. To evaluate the agreement between raters, Cohen’s kappa (k) was used as a measure of inter-rater agreement in the patterns’ classification. This analysis aimed to assess the consistency of visual ratings among the different physicians. We performed receiver-operating-characteristic (ROC) analyses to compare the discriminative power of IMT in differentiating between AD-like and LATE-like subjects obtained by visual classification. Differences in cognitive trajectories between groups were assessed using linear mixed-effects models, which included participant-specific intercepts and slopes, correcting for age.

All analyses were performed using R, version 4.0.2 (https://www.r-project.org/). A *p*-value of 0.05 was considered the significance threshold for all analyses.

## Results

Demographic and clinical data for our cohort are displayed in Table 1.

**Table 1:** Demographic and clinical features of the sample

	Negative	AD-like pattern	LATE-like pattern	Others	statistic
N	25	25	12	8	
Age	73.7 ± 4.6	68.5 ± 6.8	75.3 ± 5.2	71 ± 10	**0.008** ^**a, c**^
Education (y)	12.6 ± 4.8	13.6 ± 5.1	11.9 ± 3.3	15 ± 2.3	0.429
Sex (F/M)	15/10	13/12	5/7	1/7	0.131
Amyloid status (-/+)	10/15	4/21	3/9	5/3	0.061
MMSE	27.1 ± 2.1	22.4 ± 4.2	25.1 ± 2.7	25.9 ± 2.9	**< 0.001** ^**a, b,d**^
FCSRT immediate recall	14.3 ± 2.2	12.1 ± 2.9	11.5 ± 2.7	12.2 ± 2.6	**0.034**
FCSRT delayed free recall	6.1 ± 4.7	4.78 ± 1.9	1.0 ± 1.4	5.3 ± 2.5	0.141
FCSRT delayed total recall	13.1 ± 3.3	11.2 ± 3.2	4.5 ± 4.1	13 ± 1	**0.001** ^**b**^
TMT - A	44.7 ± 8.2	70.9 ± 40.6	42.5 ± 15.9)	79.8 ± 62.1	**0.049** ^**a**^
TMT - B	123 ± 70.4	170 ± 94.2	92 ± 37.5	137 ± 84.7	0.349
Semantic fluency	15.8 ± 3.72	10.2 ± 3.6	19.2 ± 1.2	13 ± 3.3	**< 0.001** ^**a, c**^
Phonemic fluency	13.9 ± 5.4	12.7 ± 5.2	22.5 ± 4.9	11.8 ± 1.7	0.091
Digit span	45.5 ± 12.1	33.3 ± 14.1	41.4 ± 21.7	35.6 ± 20.5	0.119

Continuous variables are reported as mean ± standard deviation and categorical variables as frequencies. *P*-values are obtained by kruskal–wallis test for continuous variables and the proportion test for frequencies. Differences between subgroups are tested post hoc with dunn test and proportion test

Abbreviations: *AD*, Alzheimer's disease; *LATE*, limbic age-related TDP-43 encephalopathy; *F*, females; *M*, males; *MMSE*, mini-mental state examination; *N*, number; *y*, year; *FCSRT*, Free and Cued Selective Reminding Test; *TMT*, Trail-Making Test

^a^AD-like is significantly different from negative

^b^LATE-like is significantly different from negative

^c^LATE-like and AD-like are significantly different

^d^AD-like is significantly different from others

^e^LATE-like is significantly different from others

### ^18^F-FDG-PET visual classification

**Fig. 1:**
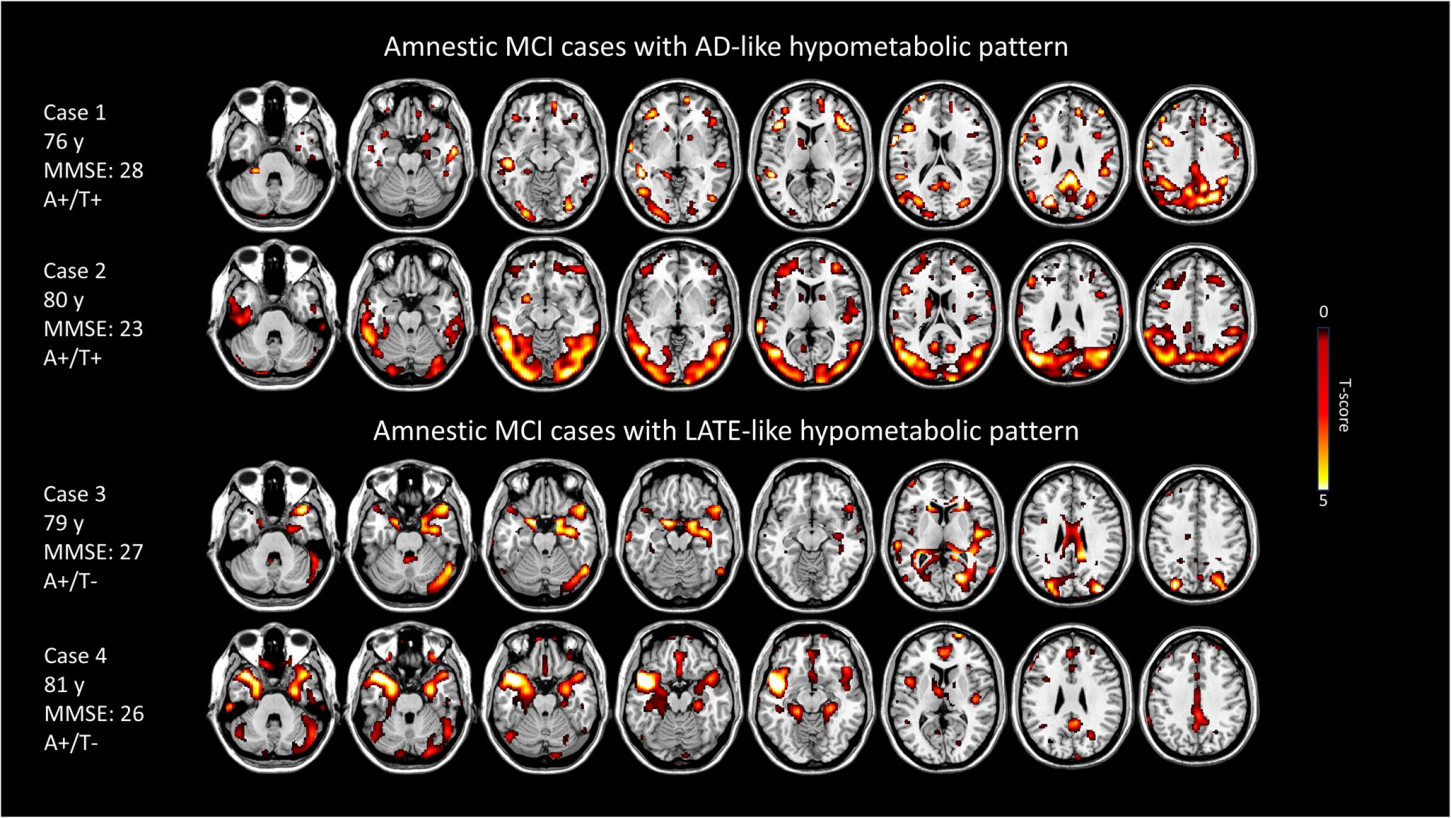
Individual examples of brain hypometabolic patterns for AD-like and LATE-like patterns. The single-subject ^18^F-FDG-PET hypometabolic patterns resulted from statistical parametric mapping (SPM) single-subject analysis versus controls; significance was set at uncorrected *p* < 0.05 at the voxel level with k > 100 voxels. Yellow/red scales represent hypometabolism severity in t-scores (*p* < 0.05, k = 100)

**Fig. 2:**
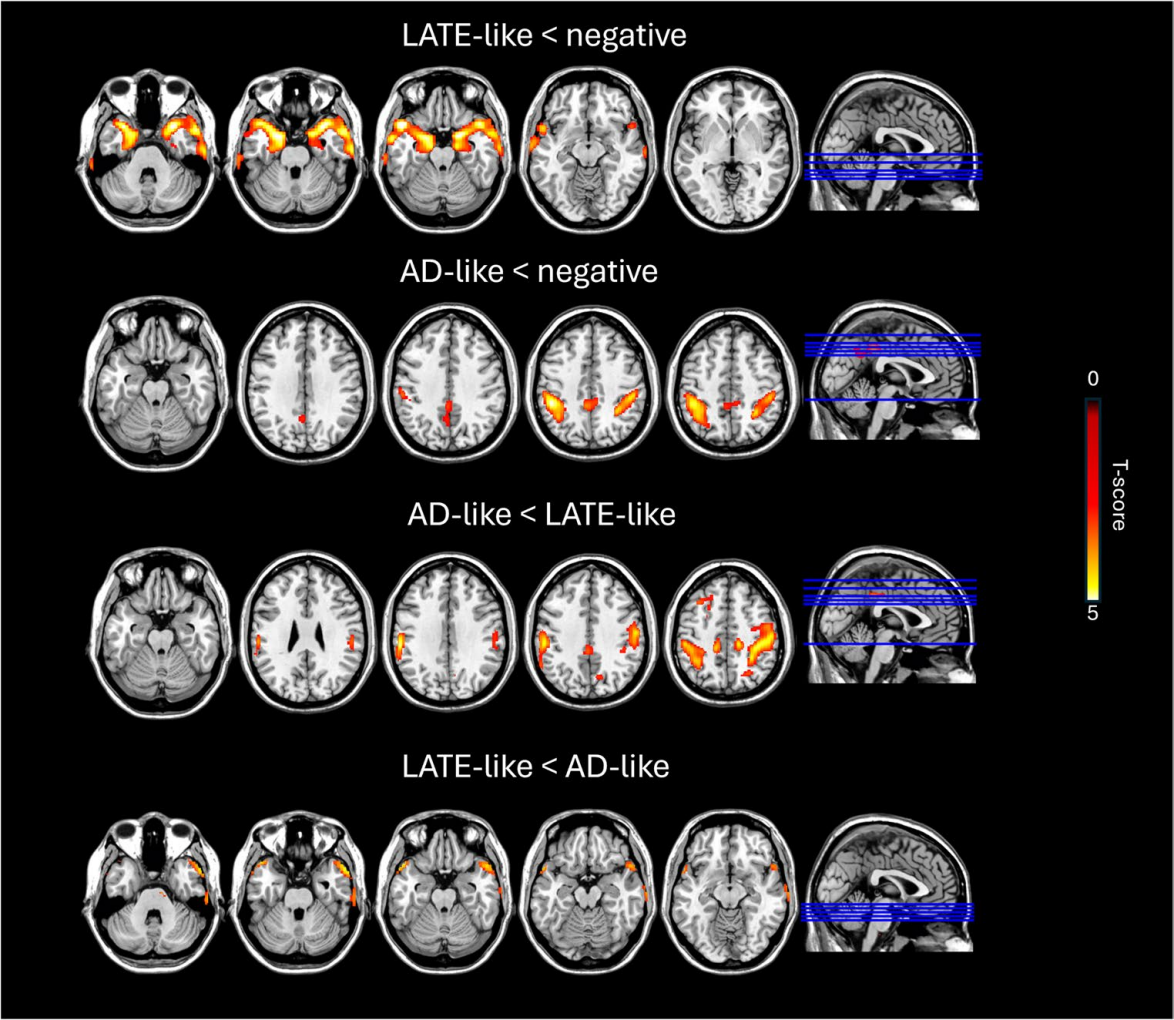
Brain hypometabolic patterns at group level and voxel-wise differences between groups. Topographical distributions of brain hypometabolism are obtained by SPM group analysis obtained by statistically comparing LATE-like and AD-like groups against negative scans and between each other. Only brain regions showed significant differences are depicted in yellow/red color scales representing hypometabolism severity (*p* < 0.005, k = 100)

Our analysis revealed a fair inter-rater reliability in the visual interpretation of ^18^F-FDG-PET single-subject maps (k = 0.40), meaning that 60% of cases were classified with the same patters by the two raters. The consensus visual rating of single-subject maps allowed the identification of two main neurodegenerative patterns: temporoparietal hypometabolism (AD-like pattern, *n* = 25), and limbic-like or medial–temporal pattern (LATE-like pattern, *n* = 12). Twenty-five subjects showed negative ^18^F-FDG scans for neurodegenerative patterns, whereas eight scans showed hypometabolic patterns suggestive of alternative degenerative conditions and were categorized as “others”. Examples of individual AD-like and LATE-like patterns are reported in Fig. 1, while Figure S2 shows all individual hypometabolic patterns of LATE-like cases. Figure 2 shows hypometabolism patterns at the group level obtained by voxel-wise group comparisons. When we compared LATE-like vs. negative scans, LATE-like patients showed significant hypometabolism in the medial temporal lobes, temporal poles and insula. Comparing AD-like vs. negative scans, AD-like patients showed significant hypometabolism in precuneus and posterior parietal regions; and a similar pattern was found comparing AD-like vs. LATE-like. Instead, LATE-like group showed more significant hypometabolism in temporal poles and insula than AD-like group.

### Clinical and biomarker differences

AD-like and LATE-like groups differed in age and semantic fluency, but not in other clinical and demographic variables (Table 1). Both patients with AD-like and LATE-like patterns had lower MMSE scores than subjects with normal scans (Table 1). AD-like patients showed worse executive function as measured by TMT– A than negative patients, whereas LATE-like patients showed impaired episodic memory as assessed by FCSRT delayed total recall than negative patients (Table 1). Regarding biomarkers, patients with LATE-like patterns had a significantly higher IMT ratio, amygdalar atrophy, and lower tau SUVR in the global ROI and all Braak stages, except for I-II, than AD-like subjects (*p* < 0.001) (Table 2). Centiloid values and hippocampal volume did not differ between AD and LATE subjects. LATE-like group differed from negative subjects only in hippocampal atrophy, that is greater in the former group (Table 2).

**Table 2:** Biomarker data

	Negative	AD-like pattern	LATE-like pattern	Others	statistic
IMT	1.3 ± 0.1	1.2 ± 0.1	1.3 ± 0.1	1.3 ± 0.1	**< 0.001** ^**a, c**^
HPV	3621 ± 400	3235 ± 500	3136 ± 439	3401 ± 614	**0.009** ^**a, b**^
MTA	0.92 ± 0.79	1.24 ± 1.00	1.88 ± 1.07	1.31 ± 1.07	**< 0.001** ^**a, c**^
Amygdala volume	1386 ± 184	1223 ± 238	1195 ± 302	1393 ± 271	**0.027**
Amygdala atrophy no/mild to moderate/severe	20/5/0 80%/20%/0%	15/9/1 60%/36%/4%	3/9/0 25%/75%/0%	4/3/1 5%/37%/13%	**0.011** ^**c**^
Centiloid	47.1 ± 45.8	67.4 ± 42.7	71.9 ± 47.4	12.7 ± 32.6	**0.017** ^**d, e**^
Global tau SUVR	1.34 ± 0.27	1.59 ± 0.42	1.32 ± 0.22	1.33 ± 0.37	**< 0.001** ^**a, c**^
Braak I-II tau SUVR	1.37 ± 0.25	1.4 ± 0.25	1.38 ± 0.2	1.2 ± 0.24	0.35
Braak III tau SUVR	1.3 ± 0.24	1.49 ± 0.35	1.32 ± 0.2	1.24 ± 0.26	**< 0.001** ^**a, c**^
Braak IV tau SUVR	1.24 ± 0.2	1.46 ± 0.34	1.21 ± 0.19	1.22 ± 0.28	**< 0.001** ^a, c^
Braak V tau SUVR	1.13 ± 0.16	1.34 ± 0.36	1.11 ± 0.11	1.1 ± 0.17	**< 0.001** ^a, c^
Braak VI tau SUVR	1.04 ± 0.12	1.14 ± 0.23	1.02 ± 0.13	1.01 ± 0.1	**< 0.001** ^a, c^

Continuous variables are reported as mean ± standard deviation

*P*-values are obtained by ANCOVA test (controlling for age and MMSE) for continuous variables. Differences between subgroups are tested post hoc with Tukey test

Abbreviations: *AD*, Alzheimer's disease; *LATE*, limbic age-related TDP-43 encephalopathy; *HPV*, hippocampal volume; *IMT*, inferior-to-medial temporal ratio; *MTA*, medial temporal atrophy; *SUVR*, standardized uptake value ratio

^a^AD-like is significantly different from negative

^b^LATE-like is significantly different from negative

^c^LATE-like and AD-like are significantly different

^d^AD-like is significantly different from others

^e^LATE-like is significantly different from others

ROC analyses were conducted to evaluate the ability of IMT to distinguish LATE-like from AD-like cases obtained by visual rating. The area under the curve (AUC) was 0.860 indicating high accuracy and, thus, a comparable performance of the visual and semiquantitative methods. Explorative ROC analyses were also conducted to evaluate the ability of IMT to distinguish LATE-like and AD-like from negative cases, resulting in fair discriminative performances (AUC_LATE−like vs. negative_ = 0.747; AUC_AD−like vs. negative_ = 0.706).

**Fig. 3:**
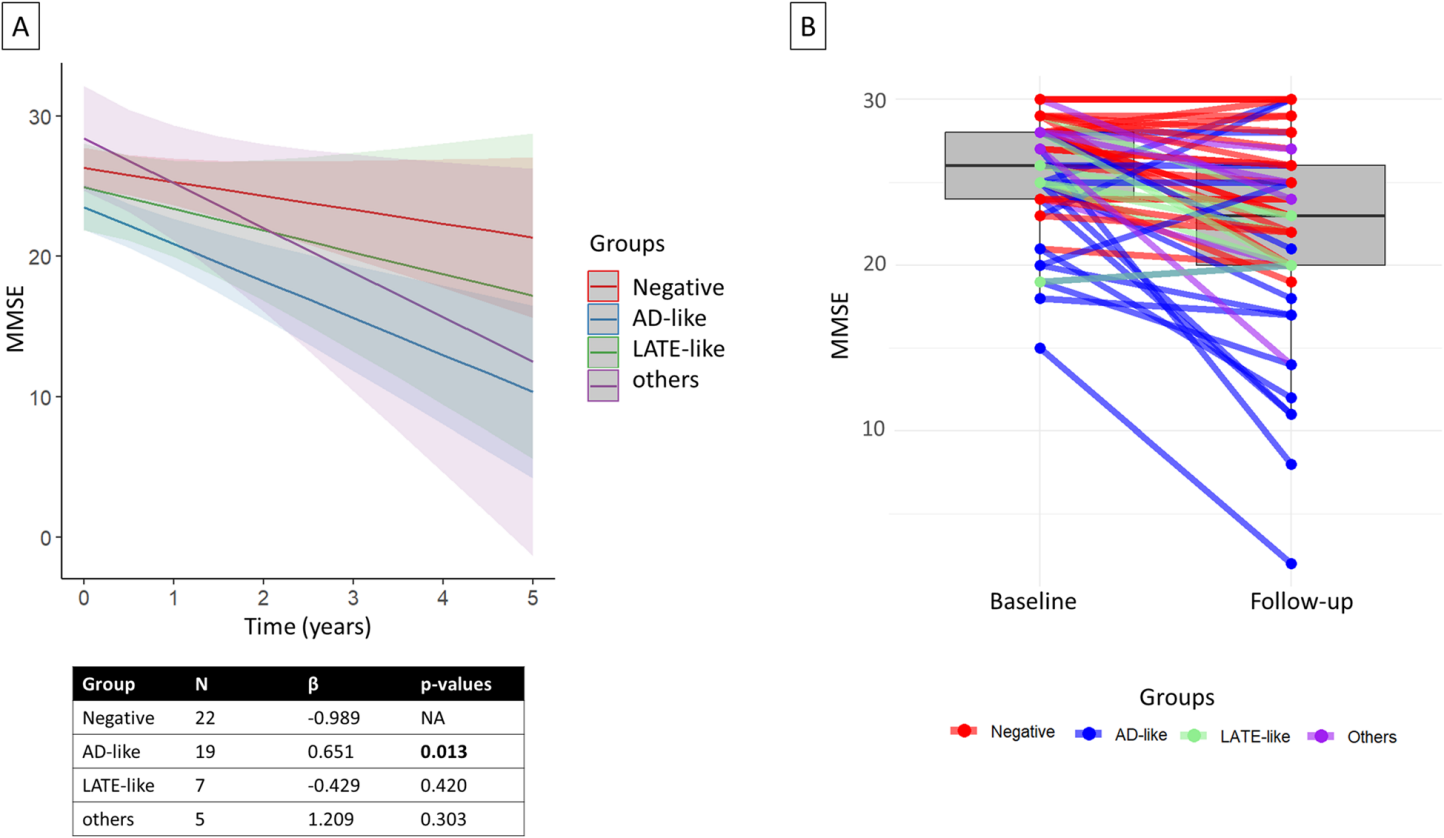
Longitudinal results. Panel **A** shows different cognitive trajectories of MMSE scores over time for the different groups visually classified based on brain hypometabolic patterns. Panel **B** shows a boxplot including individual data points for baseline and follow-up MMSE scores with lines joining paired points (last follow-up available was considered)

Linear mixed-effect models indicated that subjects with AD-like patterns (*n* = 19 with follow-up) had a faster cognitive decline than negative subjects (*p* = 0.01) whereas subjects with LATE-like pattern (*n* = 7 with follow-up) did not show any significant difference from negative subjects (*n* = 22 with follow-up) (Fig. 3).

## Discussion

Despite the promising role of ^18^F-FDG-PET in distinguishing patients with LATE-NC, no studies have yet tested the ability of the visual assessment to detect the specific temporo-limbic patterns at the individual level in a real clinical setting. In previous literature, the temporo-limbic hypometabolism and absence of neocortical degenerative pattern has been consistently associated with the presence of TDP-43 pathology at postmortem [23] and it was able to distinguish the autopsy-confirmed cases with comorbid TDP-43 from AD at group level [8, 9]. In our study, we demonstrated the possibility of visually distinguishing the specific LATE hypometabolic pattern in individuals with aMCI, which can identify a subgroup of subjects distinct from AD and controls in terms of clinical severity, medial temporal atrophy, neocortical tau load, and cognitive decline.

In our study, we identified 17% aMCI patients with LATE-like hypometabolic pattern at ^18^F-FDG-PET, in line with previous literature where LATE pathology has been observed as predominant pathology in approximately 20% of all persons beyond age 85 years with amnestic cognitive impairment [7]. The most recent clinical criteria have estimated LATE pathology, both with and without concomitant AD, to affect > 10% of all individuals beyond age 65 years and ≈ 25–40% of those beyond age 85 years [24]. Despite the same amnestic phenotype characterizing our cohort, patients showing a LATE–like ^18^F-FDG-PET pattern were significantly older and showed less tau accumulation in PET images than AD-like patients. In line with previous studies showing less abnormal amyloid and tau CSF biomarkers [9] and negative tau-PET scans [8, 25], patients in our cohort with a LATE-like pattern showed significantly less tau load in AD-related regions than AD-like cases, but no differences in amyloid. The absence of fully positive AD biomarkers in amnestic individuals strongly increases the likelihood of LATE [7]. Although the presence of amyloid at PET in patients with LATE-like patterns suggested the possibility of AD copathology, the link between amyloid positivity and a diagnosis of AD diminishes with age, and autopsy studies show substantial amyloid buildup in the brain of older individuals who did not have dementia before death [26]. According to the recent LATE clinical criteria, both amyloid and tau positivity are needed to define AD copathology [24]. Regarding tau, the lower burden at group level in LATE-like subjects hampers an AD etiology, that is instead strongly supported by the presence of neocortical tau in AD-like cases. Looking at individual cases, we found half of LATE-like patients with tau-positive scans suggesting AD copathology and the other half being tau-negative (50% with tau limited in the MTL) suggesting LATE predominant pathology. However, in the absence of follow-up tau imaging, we cannot exclude that LATE-like patients will develop tau in their brains. Longitudinal studies incorporating serial tau imaging are necessary to clarify the progression of pathology and differentiate LATE from early or atypical AD. By assessing the hypometabolism of the medial temporal lobe relative to the metabolic sparing of the inferior temporal gyrus (IMT ratio), previous studies identified subgroups of patients who were tau-negative and either amyloid-positive or negative on PET. Amyloid- and tau-positive patients with AD showed greater inferior temporal hypometabolism and, thus, a lower IMT measure compared to the tau-negative group that was thought likely to have LATE-NC with hippocampal sclerosis [8]. When we tested the IMT ratio in our cohort, we found significantly lower IMT in AD-like than in LATE-like and a good IMT performance of distinguishing visual-based subgroups (AUC = 0.86), confirming that visual and semi-quantitative approaches yield consistent outcomes. Although the IMT ratio did not differ between LATE-like and negative scans, IMT ratio was able also to distinguish between them, even if with a lower accuracy (AUC = 0.75) than the discrimination between LATE-like and AD-like. An elevated IMT ratio in absence of other degenerative patterns has been included in LATE criteria as additional supportive neuroimaging feature [24] that may offer additional accuracy in classification. However, the lack of validated thresholds limits its use in clinical settings calling for future studies setting cut-offs based on postmortem validation. Validated IMT ratio thresholds, based on large, pathologically confirmed datasets, would facilitate IMT ratio’s clinical adoption.

Subjects with LATE-like hypometabolism differed from negative scans only in medial temporal atrophy, that has been frequently associated with TDP-43 pathology and hippocampal sclerosis [7] which seemed to contribute more to hippocampal volume loss than AD [27]. Hippocampal sclerosis implied significant pyramidal cell loss and gliosis in the hippocampal formation and it is associated with severe hippocampal atrophy in elderly persons with dementia [7]. Autopsy studies showed that subjects with advanced age and hippocampal sclerosis often have TDP- 43 proteinopathy [28–31] and subjects with brain TDP-43 and without hippocampal sclerosis are a subset of LATE-NC that represent 5–40% of research subjects in autopsy series [3]. LATE-NC has been reported in the brain of individuals with AD [32], and another study reported 74% of frequency for the coexistence of both pathologies [33], suggesting that LATE-NC is not an inevitable sequela of severe AD. Other copathologies impossible to detect in vivo may coexist with LATE-NC, such as primary age-related tauopathy (PART) [34, 35], and argyrophilic grains or glial tauopathy [36–38]. However, the implications of comorbidities and various pathologies in the context of LATE-NC are still incompletely understood.

Focusing on clinical trajectories, patients with LATE-like patterns showed an overall slower disease course. We could not demonstrate significant differences in cognitive decline in LATE-like patients compared to AD-like and negative scans probably due to the small sample size and the heterogeneous aetiologies characterizing this group. Previous studies showed a faster cognitive decline in individuals with pure LATE-NC than those that had neither LATE-NC nor AD [39]. However, individuals with LATE-NC had a more gradual clinical decline than pure AD [39–41], consistent with our result showing that only AD-like patients deteriorated significantly faster than negative subjects.

Among the limitations, we are aware that the inter-rater agreement observed in our study was only fair, limiting its applicability in clinical practice, that we tried to compensate applying a consensus rating. However, our primary aim was testing the clinical translation of LATE-like patterns identified in research in a clinical cohort. Standardized rating protocols and structured training programs could improve the consistency of visual PET interpretation, and the implementation of automated or AI-assisted classification tools can be explored with the aim of enhancing objectivity and reproducibility. In addition, we acknowledge a relatively small sample size and the lack of postmortem examination, which hampers a conclusive analysis of aetiology and copathologies. Larger cohorts with autopsy confirmation are needed to refine imaging criteria and strengthen the diagnostic framework for LATE. Although in vivo biomarkers for LATE, including biofluids or PET ligands, are currently in development, they do not seem to be on the near-term horizon given also the obstacles posed by intracellular location and small pathological burden of TDP-43 proteinopathy [24, 42]. Novel radiotracers that bind to TDP-43 as well as detection methods of TDP-43 in biofluids are under investigation but they still remain an unmet need [43, 44]. Future studies should focus on developing and validating specific in vivo biomarkers for TDP-43, including PET ligands and biofluid-based assays.

In the absence of specific biomarkers for TDP-43 pathology [43], our results support the clinical utility of ^18^F-FDG-PET, a widely accessible technique, as a biomarker for helping detecting cases with LATE-NC and differentiating them from healthy aging and AD pathology in clinical settings. In both amyloid positive and negative cases, LATE-like ^18^F-FDG-PET patterns may lead to suspicion of LATE-NC. In the absence of tau, LATE can be the primary driver and a negative tau-PET might be enough to rule-out AD aetiology when the core clinical features of LATE are satisfied [24]. On the other hand, tau-PET positivity is consistent with the high frequency of LATE pathology in AD brains and suggestive of mixed pathology. In this scenario, ^18^FDG-PET can be a useful support to identify LATE-like patients, at least until specific TDP-43 biomarkers are developed. With the advent of anti-amyloid treatments for AD, the correct identification of individuals with LATE-NC represents a point of utmost importance. Clinical trials’ outcomes can be influenced by co-pathology and the potential effects of anti-amyloid therapy in mixed pathology should be investigated aware that LATE-NC is a prevalent pathological comorbidity. LATE clinical criteria [24], including ^18^F-FDG-PET as supportive biomarker, can inform about LATE (co)pathology and it would be possible investigating whether anti-amyloid agents will show similar benefits in persons with LATE-NC concomitant to AD.

## Electronic supplementary material

Below is the link to the electronic supplementary material.

Supplementary Material 1

## Data Availability

Anonymized data used in this study are available upon reasonable request from the corresponding author (VG).
