# Supplementary material for: Clinical and molecular correlates of limbic age-related TDP-43 encephalopathy (LATE) 18F-FDG-PET pattern in amnestic mild cognitive impairment
Source: Eur J Nucl Med Mol Imaging. 2025 Jun 13;52(13):5075–84. doi: 10.1007/s00259-025-07395-9 (PMC12589272; doi:10.1007/s00259-025-07395-9)
Supplement: Supplementary file 1 — Supplementary Material 1 [file 259_2025_7395_MOESM1_ESM.docx]

**Supplementary Materials**

**Figure S1.** Study Design Flowchart


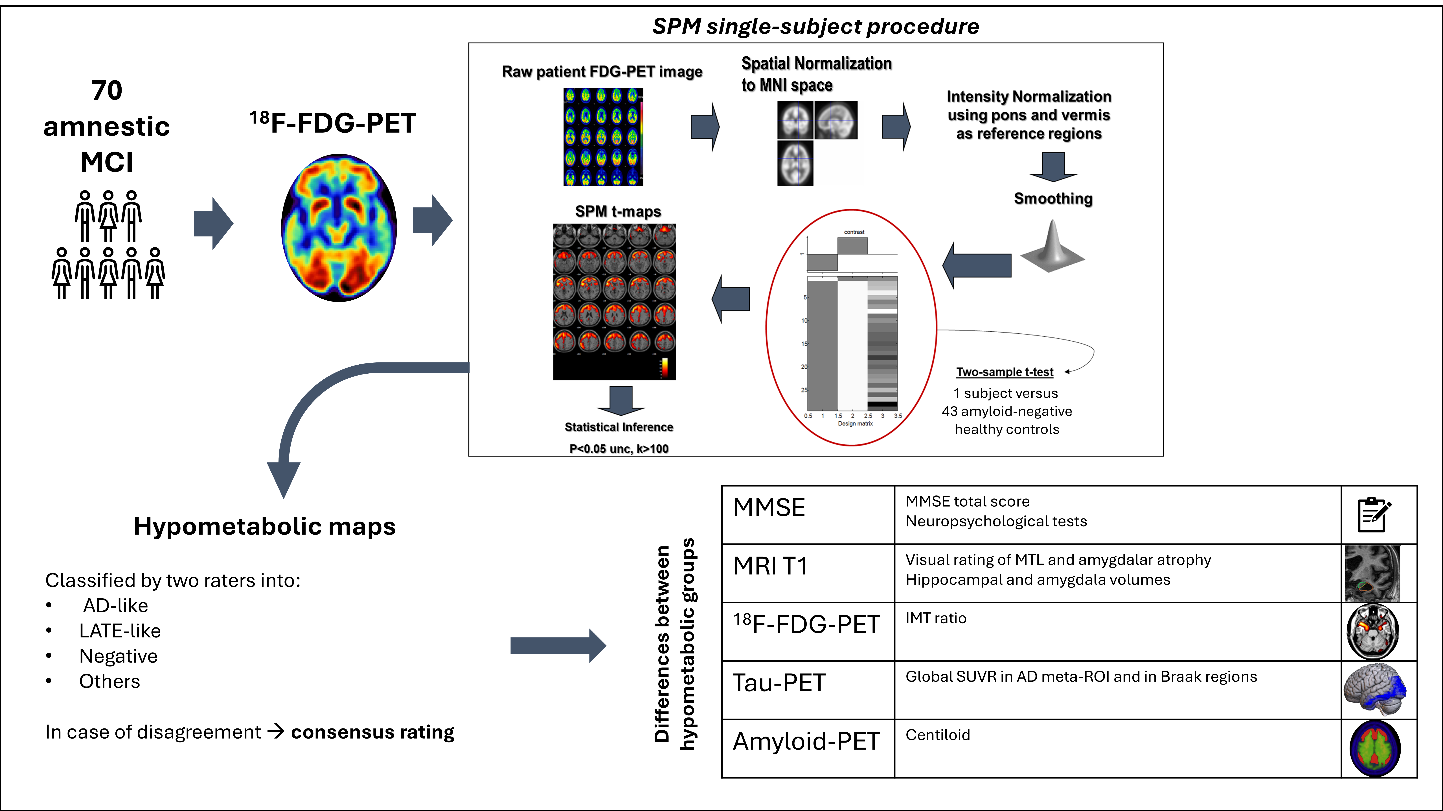


**Figure S2.** Individual cases of LATE-late hypometabolic patterns. The single-subject 18F-FDG-PET hypometabolic patterns resulted from statistical parametric mapping (SPM) single-subject analysis versus controls; significance was set at uncorrected p < 0.05 at the voxel level with k > 100 voxels. Yellow/red scales represent hypometabolism severity in t-scores (p < 0.05, k = 100). Age, MMSE and amyloid and tau status are reported for all cases. Panel A shows tau-negative cases, while Panel B shows amyloid- and tau-positive cases who thus harbor AD copathology.


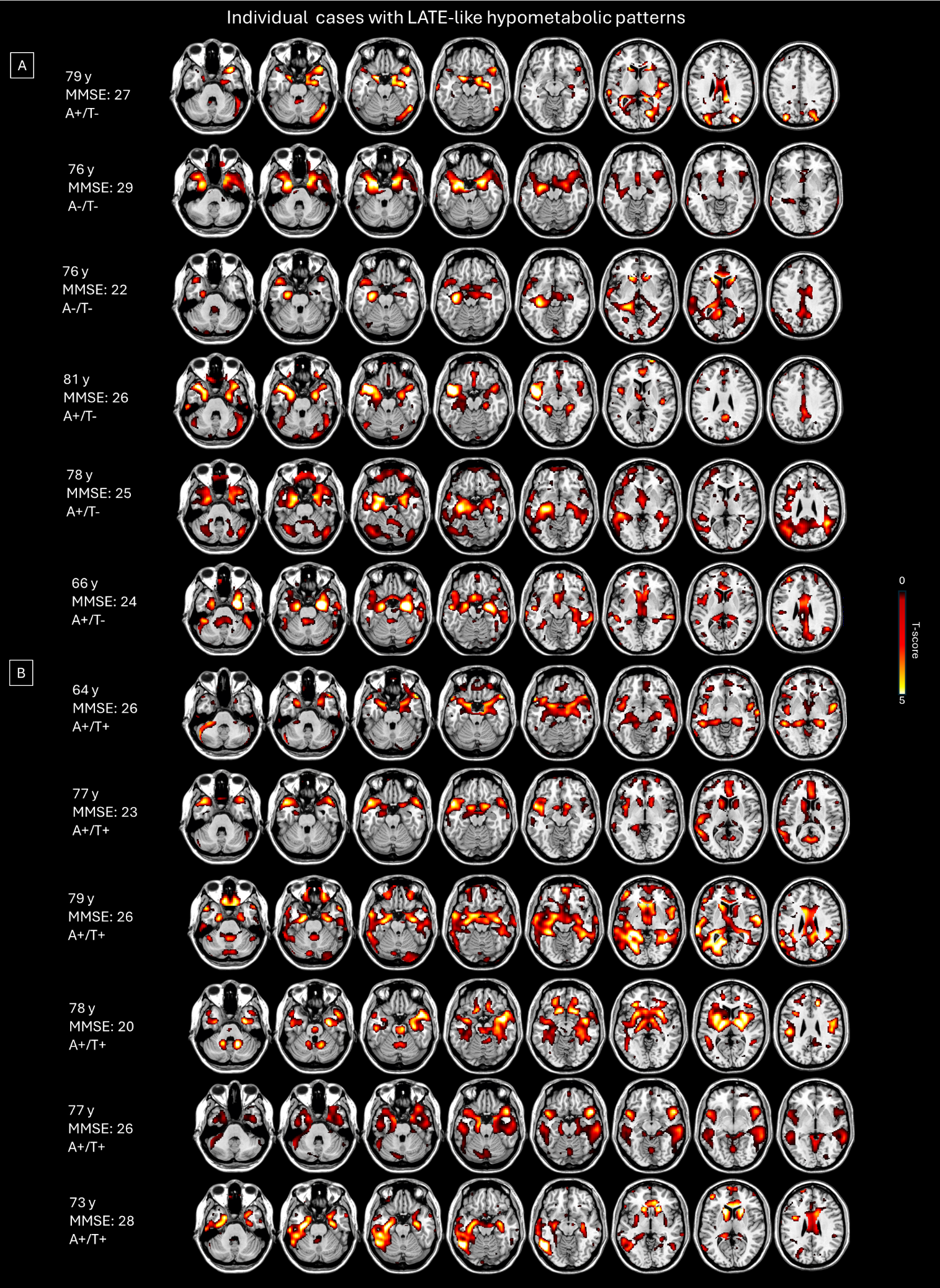


**Table S1.** The contingency table reports the visual classification of ^18^F-FDG-PET images by the two raters

|  | **Rater 1** | | | | |  |
| --- | --- | --- | --- | --- | --- | --- |
| **Rater 2** |  | Negative | AD-like | LATE-like | other | Total Rater 2’s classification |
|  | Negative | 12 | 7 | 3 | 3 | 25 |
|  | AD-like | 2 | 24 | 4 | 1 | 31 |
|  | LATE-like | 1 | 3 | 5 | 0 | 9 |
|  | other | 1 | 2 | 1 | 1 | 5 |
|  | Total Rater 1’s classification | 16 | 36 | 13 | 5 |  |
| Abbreviations: AD= Alzheimer disease, LATE= limbic age-related TDP-43 encephalopathy | | | | | | |
